# Post-COVID-19 travel behaviour patterns: impact on the willingness to pay of users of public transport and shared mobility services in Spain

**DOI:** 10.1186/s12544-021-00476-4

**Published:** 2021-03-10

**Authors:** Samir Awad-Núñez, Raky Julio, Juan Gomez, Borja Moya-Gómez, Julián Sastre González

**Affiliations:** 1 Department of Civil Engineering, Universidad Europea de Madrid, Villaviciosa de Odón, Spain; 2 Centro de Investigación del Transporte (TRANSyT), Universidad Politécnica de Madrid, Madrid, Spain; 3 Instituto de Movilidad, Sevilla, Spain

**Keywords:** Post-COVID-19 mobility, Post-COVID-19 travel behaviour, Heckman modelling, Discrete choice modelling

## Abstract

**Background:**

The COVID-19 crisis has meant a significant change in the lifestyle of millions of people worldwide. With a lockdown that lasted almost three months and an impulse to new normality, transport demand has suffered a considerable impact in the Spanish case. It is mandatory to explore the effect of the pandemic on changes in travel behaviour in post-COVID-19 times.

**Methodology:**

A nationwide survey was carried out during the lockdown in Spring 2020 to overview the recent changes. The survey collected both stated preferences (socio-demographic characteristics and mobility-related attributes), and revealed preferences (individuals’ habits, especially in the frequency of the trips according to the trip purpose, and opinions regarding the willingness and acceptability of these changes, and which actors would have to drive them, and how) of individuals. This paper aims to study and understand the willingness to adopt a set of measures to improve the safety conditions of public transport and shared mobility services against possible contagion from COVID-19 and the willingness to pay for them.

**Results:**

The results obtained show that some measures, such as the increase of supply and vehicle disinfection, result in a greater willingness to use public transport in post-COVID-19 times. Similarly, the provision of covers for handlebars and steering wheels also significantly increases individuals’ willingness to use sharing services. However, respondents expect that these measures and improvements would be implemented but maintaining the same pre-COVID-19 prices. The results of this research might help operators deploy strategies to adopt their services and retain users.

## Introduction and state of knowledge

The lifestyles of a large worldwide population have abruptly changed during the first half of 2020. The COVID-19 pandemic, caused by the virus SARS-CoV-2, is the first pandemic in decades that nearly stopped the world, and its most adverse impacts are yet to be seen further in time.

The first confirmed cases appeared in Wuhan, China, in December of 2019. Despite the local government’s efforts to contain the disease, it rapidly extended worldwide [23, 26]. By January 30, 2020, the World Health Organization (WHO) declared COVID-19 as a Public Health Emergency of International Concern [35]. By March 11, 2020, the WHO updated its status, declaring it as a worldwide pandemic [36].

Governments had to deploy severe measures to contain the virus spreading and try to flatter the incidence curve. The standard strategy laid on two main fundamentals: to restrain mobility and promote social distancing. Mobility restrictions were applied at different levels, at local and regional ones (e.g., restraining the length of walking or motorised displacements), and at national and even international levels (closure of entire regions [10]), as the burden of COVID-19 patients collapsed hospitals on the most severely affected countries.

Social distancing in Western Europe was positively encouraged, when not mandatory. In Spain, one of the most affected countries by COVID-19 during spring 2020, a mandatory lockdown was decreed on March 15 to avoid physical-social interaction by closing schools and most of the economic activities and allowing citizens mobility only to a few activities, such as going to the grocery or access to medical care [8]. Moreover, some Spanish regions enabled preventive measures before the mandatory lockdown. For instance, the region of Madrid enacted closing schools on March 11 [7], and almost all of them recommended reducing physical-interactions. These measures, known as non-pharmaceutical Interventions (NPIs) were already used on previous airborne pandemics, such as influenza outbreaks [1, 29] and their effectiveness was evaluated by Jefferson et al. [22] or Fong et al. [16]. Some recent studies assess the effectiveness of these measures on COVID-19 [9, 27].

During the lockdown in Spain, mobility to workplaces dropped 80% compared with pre-COVID-19 trends [17, 28]. The most affected mode was public transport rather than private cars [4]. Moreover, Spain reported the lowest vehicle miles travelled (VMT) in Europe, with only 12% of the pre-COVID-19 VMT during the second week of April [21]. The lockdown in Spain was partially loosened by the end of May, and a period of “new normality” started, in which the most severe measures were slightly eased, but preserving some fundamentals of the NPIs.

Due to this extreme change in people’s habits, the COVID-19 pandemic may have pervasive effects on the way people interact and travel later on. The implications of social distancing might be drastic. It can be expected that people will travel less and try to avoid public transport [14]. Shared options such as car-sharing, moped scooter-sharing, bike-sharing, or ride-hailing may be less attractive, given the fear of exposure to the virus [20]. Meanwhile, walking and private cycling might gain particular importance [32]. To keep users, operators of public transport and shared mobility services will have to invest resources in biosecurity measures [15] to satisfy governmental requirements and give users a higher safety sensation that encourages their use. Many questions arise on individuals’ willingness and acceptability of these measures, which actors should impulse them, and how.

Willingness To Use (WTU) and Willingness To Pay (WTP) are highly relevant concepts for managers and academics, as WTP is the central input for pricing and decision making [33] In the field of transportation, WTP has been widely studied in many different aspects, such as public transport crowding reduction [25], road safety [30], and facilities improvements [31], among others.

As noted in Haghani et al. [18], the COVID-19 *“has triggered an avalanche of scientific research, both within and outside the medical domain”.* Even though the information generated is vast, this exhaustive analysis of COVID-19’s scientific publications has evidenced the lack of research on the effects of the COVID-19 pandemic on transportation. Previous studies have mainly focused on the relationship between transportation and disease spread processes, such as modelling and simulation, both on short-scale commuting flows [12] and long-distance airline traffic [6]. A research gap may then be identified regarding the study of WTU and WTP for COVID-19-safety measures on urban transportation services. In this context, this paper explores individuals’ acceptability towards a set of generic measures related to biosafety in the field of mobility in Spain, such as the mandatory use of face masks, gloves, intensive disinfection protocols, and their influence on the use of different shared transport modes.

This research topic is of particular importance due to the imminent reduction in transport demand in the short-term after the lockdown. Then it will be crucial for transport operators to deploy strategies and measures to retain users and reverse the negative tendency, first being aware of the WTU the available transport options and then understanding the WTP for these measures. To that end, we conducted a nationwide survey in Spain, which was carried out during the country’s lockdown period.

This paper is structured as follows. Section 2 shows the methodology, the modelling approach, and the first findings of the descriptive analysis of the survey. Section 3 presents modelling results and discussion. Finally, Section 4 sets out the main conclusions of the research.

## Methodology

This paper explores individuals’ willingness to use and to pay for using public transport and shared mobility services given a set of COVID-19 safety measures to be implemented after the lockdown. Due to the particular circumstances during lockdown conditions, neither face-to-face focus groups nor interviews were feasible. Therefore the required information was collected via online surveys, from April 28, 2020.

### Survey

The survey was structured into three main sections: (1) socio-demographic information, (2) mobility behaviour in pre-COVID-19 pandemic times, and (3) the possible user adoption of post-COVID-19 adaptations of public transport and transport sharing services.

The survey included questions regarding both Revealed Preferences (RP) and Stated Preferences (SP). Firstly, RP questions characterise individuals’ socio-demographic and mobility behaviour, including travel frequency according to the purpose of the trip, and the transport mode was chosen for each particular purpose. These questions refer to the situation before and after the lockdown. Secondly, SP questions foresee the changes in the habits and collect respondents’ opinions towards some potentially implemented measures. These measures are related to possible post-COVID-19 adaptations of public transport and shared mobility services (car-sharing, moped scooter-sharing, bike-sharing, and kick scooter-sharing) and hailing services (including ride-hailing and taxi). Therefore, these questions capture individuals’ acceptance of each measure and their willingness to pay if they involve an extra cost that the transport operator transfers to the user. Finally, some control questions are also included to ensure that responses are coherent throughout the questionnaire. The information collected is exploited to conduct a modelling approach to design policies directed at maintaining the sustainability of the transport system. Table 1 shows the set of variables collected in the survey.

**Table 1:** Explanatory Variables collected in the research

Part of the survey	Categories
Respondent profile	
Age	16–29, 30–49, 50–64, 65 or more
Gender	Male, Female, No answer
Residential location	Open response
Net monthly salary [Euro]	Below 500; 501–1000; 1001–1500; 1501-2000; 2001-2500; 2501-3000; Above 3000
Occupation of respondent	
Occupation before COVID-19	Unemployed, Student, Houseworker, Retired/pensioner/disabled, Worker, Worker, and Student, Others
Expected occupation after COVID-19	Unemployed, Student, Houseworker, Retired/pensioner/disabled, Worker, Worker, and Student, Others
Working mode	Face-to-face, Teleworking
Respondent travel	
Frequency of travel by mode of transport before COVID-19	Intensive (once per month or less), Non-intensive (more than once per month)
Travel frequency according to the reason for travel before COVID-19	Intensive (once per month or less), Non-intensive (more than once per month)
Expected frequency of travel by mode of transport after COVID-19	Intensive (once per month or less), Non-intensive (more than once per month)
Expected travel frequency according to the reason for travel after COVID-19	Intensive (once per month or less), Non-intensive (more than once per month)
Reason to change or maintain the mode of transport	Possibility of contagion, the supply of public transport, car ownership and driving license, congestion, price, environmental reasons
Potential changes	
Use of public transport	If the supply increases so that vehicles are less crowded, If vehicles are cleaned and disinfected daily, If masks, gloves, or hydroalcoholic gels are provided with each use, If only those not contracting/positive for COVID-19 are certified to use the service, Other measures
Use of car-sharing	If masks, gloves, or hydroalcoholic gels were provided with each use, If the vehicles were cleaned and disinfected daily, If it was certified that none of the customers of the day had tested positive for COVID-19 through the service app, If it was certified that only those who had not contracted/positive for COVID-19 could use the service, Other measures
Use of ride-hailing	If masks, gloves, or hydroalcoholic gels were provided with each use, If the vehicles were cleaned and disinfected daily, If it was certified that none of the customers of the day had tested positive for COVID-19 through the service app, If it was certified that only those who had not contracted/positive for COVID-19 could use the service, Other measures
Use of bike−/scooter-sharing	If they give masks, gloves, or hydroalcoholic gels with each use, If they use helmets that do not have contact with mouth, nose, and eyes, If it is certified that none of the customers of the day has tested positive in COVID-19 through the app of the service, If it is certified that only those who have not contracted/tested positive in COVID-19 can use the service, If they give covers for handlebars and steering wheels, Other measures (specify)
Use of moto sharing	If they give masks, gloves, or hydroalcoholic gels with each use, If they use helmets that do not have contact with mouth, nose, and eyes, If it is certified that none of the customers of the day has tested positive in COVID-19 through the app of the service, If it is certified that only those who have not contracted/tested positive in COVID-19 can use the service, If they give covers for handlebars and steering wheels, Other measures (specify)
Extra cost willing to pay	0%, up to 5%, up to 10%, up to 15%,... up to 100%

The survey was open to receive answers for two weeks, and 984 respondents participated with valid responses. We considered valid responses if the respondent would completely answer that subsection of the survey. All valid responses are used in section 3.

### Heckman model

We adopt a choice modelling framework based on Heckman specification to explore individuals’ willingness to use and willingness to pay regarding specific transport modes in post-COVID-19 time. Five different transport options were included in the questionnaire: i) public transport, ii) car-sharing, iii) taxi/ride-hailing, iv) bike-sharing/kick scooter-sharing, and v) moped scooter-sharing. They all imply transport options in which cleanliness and sanitising would be managed by the operators, either public or private.

In this respect, willingness to pay values were only reported by the subsample of individuals who were willing to use a specific transport mode. According to Heckman [19], if a dependent variable is estimated just from a set of nonrandom observed values, thus modelling estimates may be biased. This fact is because there is a correlation between the errors that determine whether a case is unobserved/missing (in our case, individuals not willing to use a specific transport option) and the errors determining the outcome variable (in our case, willingness to pay). Heckman [19] proposed a two-step method in order to correct the problem of sample selection bias. Below, its most essential aspects are outlined.

Heckman approach is a two-equation model, eq. 1: selection, and eq. 2: outcome. First, willingness-to-use a specific mode of transport (*Y*_n_) is a binary variable modelled in the selection equation. It indicates whether each case in the sample is observed or unobserved. Particularly, Y_n_ = 1 if the individual n would be willing to use a specific transport mode in post-COVID-19 times; 0 otherwise. Second, the dependent outcome variable to be estimated is *y*_*n*_ (willingness to pay for using a specific transport mode in post-COVID-19 times), which is an ordinal variable in our model. Willingness to pay was collected in the questionnaire, and is expressed in the model in relative terms (that is, percentage increase over current prices) to adapt to the different levels of prices and to purchase power across metropolitan areas and regions in Spain. Data on *y*_*n*_ are only available if *Y*_*n*_ > 0. Both variables y_n_ and Y_n_ are jointly modelled in the Heckman procedure to handle the sample-selection problem.

In the first step (eq. 1: **selection**), a binary probit regression is performed with the whole sample to determine the likelihood of Y_n_ being observed (*Y*_*n*_ > 0):

1 $$ {Y}_n=\mathbf{1}\cdotp \left({\upgamma}_p\ {\mathrm{X}}_{np}+{\mathrm{E}}_n>0\right) $$

where *X*_*np*_ are vectors of regressors for the selection equation, *γ*_*p*_ are vectors of parameters to be estimated, **1(·)** is the indicator function, and E_n_ is a random-error term.

In the second step (eq. 2: **outcome**), the modelling coefficients are calculated using the sample’s portion with observed values for the dependent variable solely (individuals willing to use a specific transport mode). An ordered probit model is adopted in terms of the probability of accepting a specific willingness-to-pay level. Then, we assume that the probability of a specific choice (the ordinal outcome y_n_ being equal to a specific value j_k_), is given by the probability that the utility of individual n (U_n_ = *β*_*p*_ *x*_*np*_) falls within τ_k_ and τ_k-1_ thresholds:

2 $$ P\left(y={j}_k\right)=\mathrm{F}\left({\uptau}_k-{\mathrm{U}}_n\right)-\mathrm{F}\left(\ {\tau}_{k-1}-{\mathrm{U}}_n\right)=P\left({\tau}_{k-1}<{\beta}_p\ {x}_{np}+{e}_n<{\tau}_k\right) $$

where τ_1,…,m_ represent the thresholds defined, x_np_ are vectors of regressors for the outcome equation, β_p_ are vectors of parameters to be estimated and e_n_ is a random-error term. The model can be also expressed as:

3 $$ y=\left\{\begin{array}{c}{j}_1\  if\ {U}_n\le {\tau}_1\\ {}{j}_2\  if\ {\tau}_1\le {U}_n\le {\tau}_2\ \\ {}\dots \\ {}{j}_{m+1}\  if\ {\tau}_m\le {U}_n\end{array}\right. $$

Furthermore, we should note that the error terms E_n_ and e_n_ have a bivariate normal distribution with mean zero and the following variance matrix:

4 $$ \varSigma =\left[\begin{array}{cc}1& \rho \\ {}\rho & 1\end{array}\right] $$

The Heckman procedure has been widely utilised for econometric analysis, mainly to calculate wage equations based on observed and unobserved wages (see e.g. [5]). In the transport field, the Heckman procedure has been used within the context of ride-sourcing platforms to analyse the two-step decisions made by drivers: to work or not, and if so, how long to work [34]. The reader is referred to as Xu et al. [37] to know more about the advantages of Heckman procedures concerning unobserved heterogeneity and selectivity bias/endogeneity problems simultaneously. Additionally, further details on the Heckman procedure for ordered categorical outcomes are provided in de Lucca & Perotti [13].

## Modelling results: willingness to use and to pay for transport services

### Demographics and uses trends

**Fig. 1:**
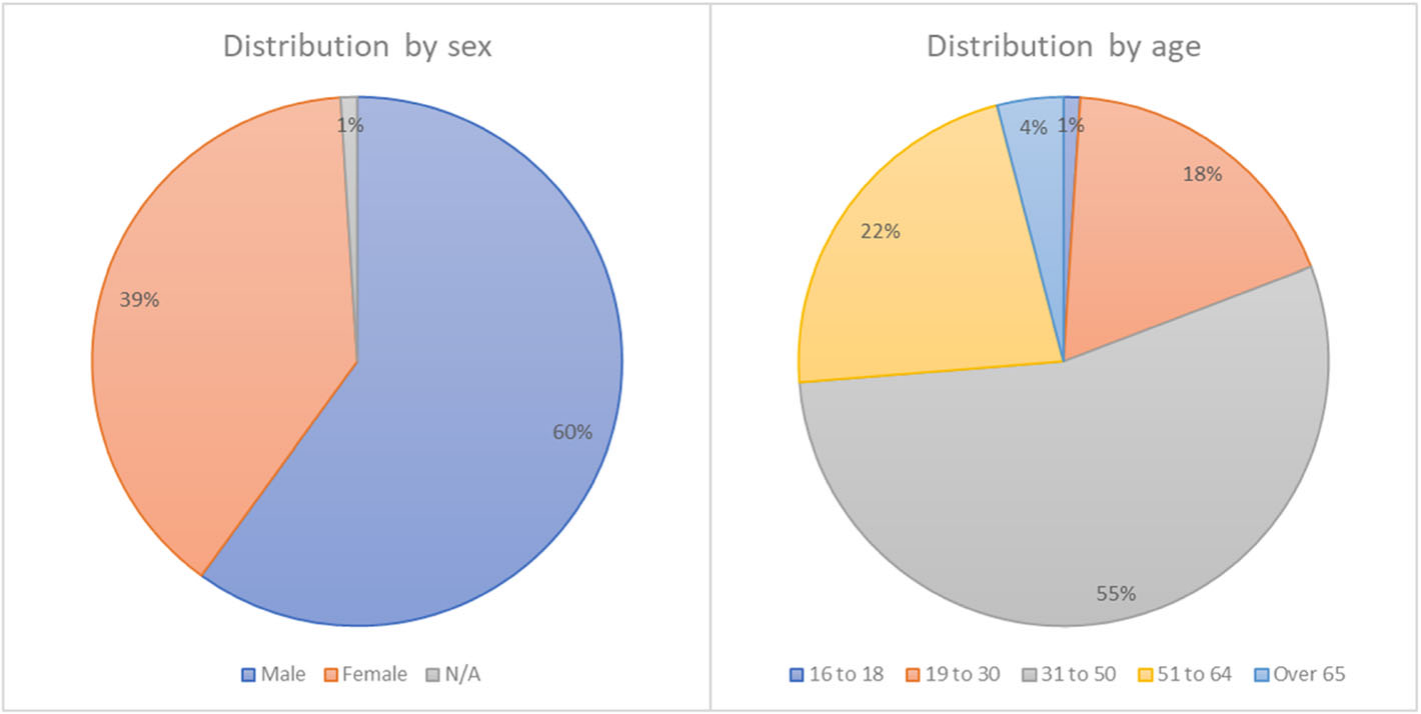
Respondents distribution by sex and age

Although the questionnaire had been opened for two weeks in Spring 2020, most of the answers were received in the first days. At that time, the lockdown was still in progress. Thus, the responses received are conditioned by the widespread paralysis of economic sectors, the high uncertainty about the working future of many workers, and the break-in classes at schools and universities. Figure 1 shows the distribution of respondents by sex and age.

**Fig. 2:**
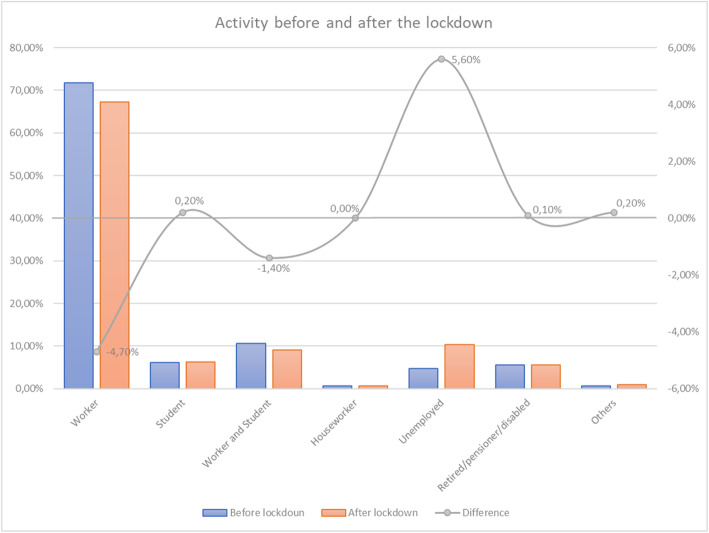
Activity before and after the lockdown

The evolution in the activity before and after the lockdown (Fig. 2) highlights that many workers expected either not to return to their jobs or telework at least a few days a week. The number of unemployed respondents is expected to double, from around 5% before lockdown to 10% after lockdown. These results have important implications for the number of trips.

**Fig. 3:**
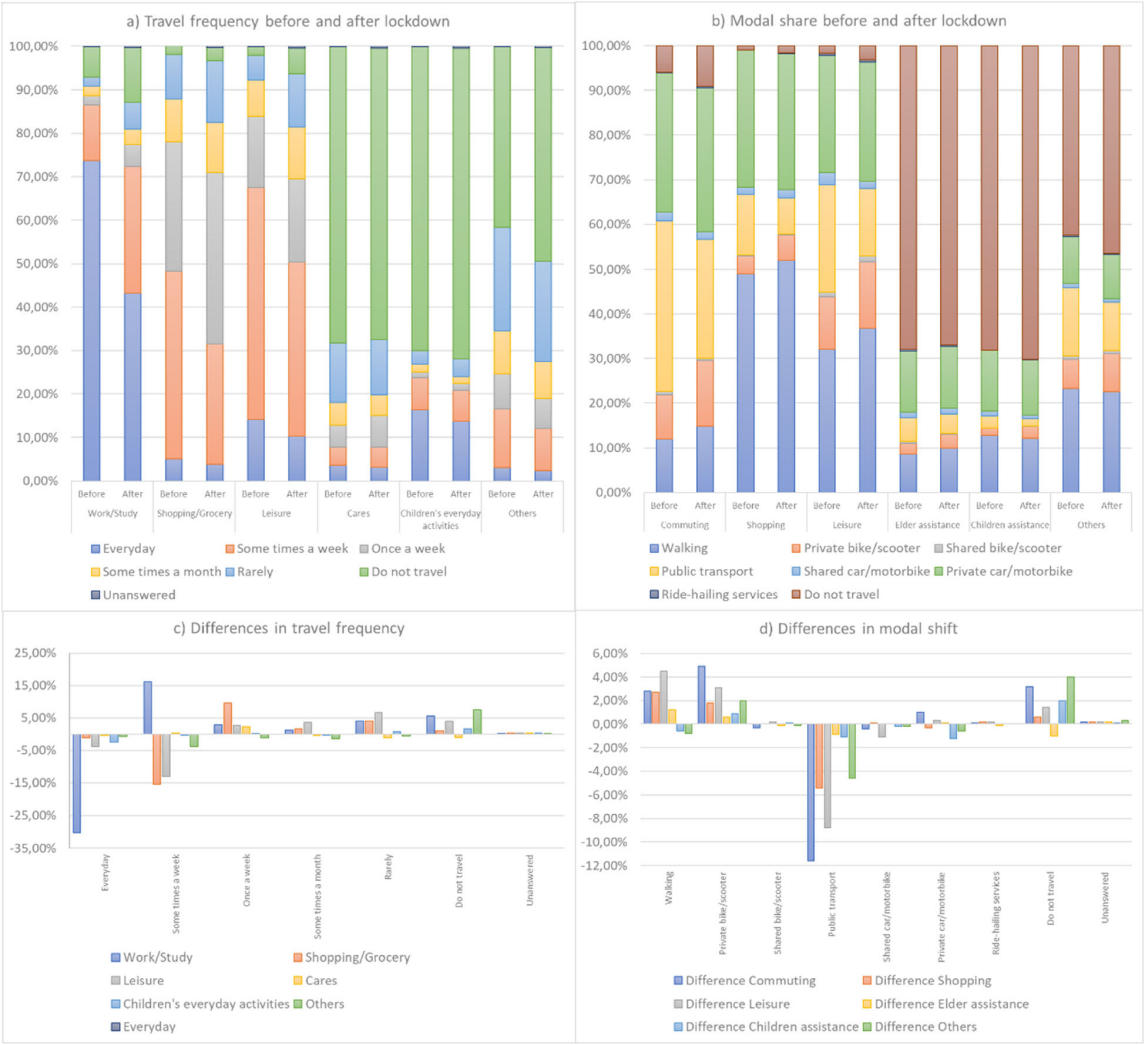
Travel frequency and modal share before and after lockdown

Regarding modality for those who expected to continue working after COVID-19 lockdown, 38% reported that they would telework, 38% that they would work in person, and 24% do not know which modality they would do so. These responses reflect the general drop in travel frequency for commuting reasons. For instance, travelling daily for commuting reasons, including going to work/education centre, decreases by 30.3%, while travelling for this reason sometimes a week increased by 16.2%. However, as can be seen in Fig. 3, travel frequency is reduced for all trip purposes. Trips related to shopping/grocery and leisure activities, which in most cases took place once a week, reduced their percentage by 15.5% and 13.1%, respectively. Respondents expected to stop making a large number of trips. The trips that they expected to be avoided the most (7.5%) are those included in the “others” category. The following reasons are “work/study” and “leisure”. In the first case, it was expected that 5.6% of the trips would not be made, while in the second case, it was thought that 3.9% would be avoided. Only travelling for “care” reasons was expected to increase by 1.2%.

According to the responses obtained, the mode of transport mostly expected to be reduced is public transport. Its use was reduced for all travel purposes, especially for “work/study” (− 11.6%), “leisure” (− 8.8%) and “shopping/grocery” (− 5.4%). By contrast, the transport modes most expected to grow were walking and private bicycles and kick scooters. In the case of walking, it mainly increases regarding “leisure” trips (+ 4.5%). In the case of private bicycles and kick scooters, they mainly grow for “work/study” (+ 4.9%) and “leisure” (+ 3.1%) purposes. Shared transport services remain fairly constant, with slight differences between + 0.20% (shared bicycle/moped scooter for commuting or leisure) and − 1.1% (shared car/motorbike for commuting or leisure).

Ride-hailing services were also expected to remain constant for all trip purposes. Private car and motorcycle would also show few changes, with the most significant changes for commuting trips (+ 1.0%) and taking care of children (− 1.2%). A preference for the use of individual means of transport is then detected, with private car and walking/bicycle/kick scooter options standing out. The main reasons reported were fear of contagion and less congestion (20% and 14% of those who change modes of transport, respectively). The reasons for changing mode and starting to walk or ride a bike or scooter were the same.

These results are reasonable, especially given the high uncertainty regarding the future employment situation of many respondents and the limited information available about the virus when the survey was launched.

### Preliminary findings

This subsection presents the main findings regarding individuals’ opinions towards the use of specific transport modes in post-COVID-19 times, namely: i) public transport; ii) car-sharing; iii) taxi/ride-hailing; iv) bike-sharing/kick scooter-sharing, and v) moped scooted-sharing. Respondents were asked whether they would use (willingness to use) a specific mode of transport if the operator would implement a specific measure. For individuals with an affirmative response were asked about their willingness to pay for these measures if they would imply an extra cost to be assumed by the user.

Preliminary findings on survey valid responses are displayed about the five modes, regarding both the whole sample (Table 2) and the subsample of potential users, i.e., individuals willing to use a specific transport mode (Table 3). As can be observed, the willingness to use transport modes in post-COVID-19 times greatly varies throughout the sample.

**Table 2:** Individuals’ willingness to use specific transport modes: distribution across socio-demographics

VARIABLES		PUBLIC TRANSPORT			CAR-SHARING			BIKE-SHARING / KICK SCOOTER-SHARING			TAXI / RIDE-HAILING			MOPED SCOOTER-SHARING		
		WILLING TO USE (POST-COVID)	WILLINGNESS TO PAY		WILLING TO USE (POST-COVID)	WILLINGNESS TO PAY		WILLING TO USE (POST-COVID)	WILLINGNESS TO PAY		WILLING TO USE (POST-COVID)	WILLINGNESS TO PAY		WILLING TO USE (POST-COVID)	WILLINGNESS TO PAY	
			ONLY WITH SAME PRICES	WITH HIGHER PRICES		ONLY WITH SAME PRICES	WITH HIGHER PRICES		ONLY WITH SAME PRICES	WITH HIGHER PRICES		ONLY WITH SAME PRICES	WITH HIGHER PRICES		ONLY WITH SAME PRICES	WITH HIGHER PRICES
Monthly Income	Below 500 Euro	97.3%	30.1%	67.1%	69.9%	28.8%	41.1%	74.0%	24.7%	49.3%	72.6%	28.8%	43.8%	56.2%	14.0%	42.1%
	500 to 1000 Euro	89.5%	31.4%	58.1%	57.0%	27.9%	29.1%	62.8%	24.4%	38.4%	73.3%	33.7%	39.5%	47.7%	19.7%	27.9%
	1000 to 1500 Euro	89.3%	26.5%	62.8%	52.6%	18.4%	34.2%	70.4%	24.5%	45.9%	65.3%	24.5%	40.8%	45.4%	20.2%	25.2%
	1500 to 2000 Euro	91.7%	25.9%	65.9%	54.6%	20.5%	34.1%	71.2%	25.4%	45.9%	67.3%	24.4%	42.9%	42.4%	17.0%	25.5%
	2000 to 3000 Euro	88.9%	21.7%	67.2%	50.0%	13.1%	36.9%	66.7%	23.7%	42.9%	72.7%	27.8%	44.9%	43.9%	13.7%	30.2%
	Above 3000 Euro	81.7%	15.9%	65.9%	51.2%	13.4%	37.8%	53.7%	18.3%	35.4%	72.0%	26.8%	45.1%	36.6%	11.1%	25.5%
Age	Under 30	94.4%	27.7%	66.7%	68.2%	25.3%	42.9%	81.2%	27.9%	53.3%	78.0%	30.4%	47.6%	62.0%	22.8%	39.2%
	30 to 49	90.3%	24.7%	65.6%	51.5%	19.4%	32.1%	68.8%	24.6%	44.2%	68.7%	28.0%	40.7%	43.8%	16.3%	27.6%
	50 to 64	84.7%	26.8%	57.9%	53.2%	19.0%	34.1%	60.0%	21.1%	38.9%	64.8%	23.5%	41.3%	35.5%	14.6%	20.9%
	Above 64	87.5%	17.5%	70.0%	42.1%	5.3%	36.8%	32.4%	13.5%	18.9%	73.7%	26.3%	47.4%	16.2%	0.0%	16.2%
Gender	Male	87.9%	24.8%	63.1%	52.3%	19.0%	33.3%	66.7%	26.0%	40.6%	66.4%	27.0%	39.4%	42.9%	17.5%	25.4%
	Female	92.8%	25.7%	67.1%	58.0%	20.7%	37.2%	68.9%	19.8%	49.1%	75.4%	27.3%	48.1%	46.8%	14.4%	32.4%
Lost employment after COVID		86.2%	40.0%	46.2%	63.1%	38.5%	24.6%	64.9%	24.6%	40.4%	58.6%	22.4%	36.2%	42.1%	16.4%	25.7%
TOTAL respondents with valid responses in the transport mode		89.7%	25.4%	64.3%	54.6%	19.8%	34.7%	67.7%	24.0%	43.7%	66.4%	26.1%	40.3%	40.6%	11.4%	29.3%

**Table 3:** Potential users’ willingness to use specific transport modes and potential measures to protect against COVID-19

Measures to protect against COVID	WILLING TO USE PUBLIC TRANSPORT		WILLING TO USE CAR-SHARING		WILLING TO USE BIKE-SHARING/KICK SCOOTER-SHARING		WILLING TO USE TAXI/RIDE-HAILING		WILLING TO USE MOPED SCOOTER-SHARING	
	% willing to use if …	% willing to pay more	% willing to use if …	% willing to pay more	% willing to use if …	% willing to pay more	% willing to use if …	% willing to pay more	% willing to use if …	% willing to pay more
Increasing supply to avoid crowding	70.6%	52.9%	30.7%	20.0%	*	*	13.3%	9.0%	*	*
Increasing cleanliness and sanitising	52.1%	40.0%	54.9%	37.5%	*	*	76.3%	48.9%	*	*
Providing masks, gloves and sanitiser gel	38.1%	27.9%	32.9%	22.6%	38.1%	27.5%	40.7%	27.6%	35.6%	22.0%
Certifying that only those non infected by COVID-19 can use the service	14.9%	11.4%	26.3%	17.6%	10.3%	6.7%	19.9%	13.6%	10.5%	7.1%
Certifying that user within the same day was not infected by COVID	*	*	*	*	14.8%	10.6%	*	*	11.5%	7.9%
Providing helmets with no contact with mouth, nose and eyes	*	*	*	*	25.3%	19.1%	*	*	38.5%	26.7%
Providing covers for handlebars and steering wheels	*	*	*	*	51.3%	36.4%	*	*	41.4%	27.7%
TOTAL potential users	843	604	501	319	593	383	624	379	382	275

Public transport is the option with the highest willingness to be used. 89.7% of individuals reported that they would use these services in post-COVID-19 times (Table 2), which seems somewhat high, given that the survey was conducted in the critical period of the lockdown. Interestingly, around 64.3% of total respondents declared that they would pay more (compared to pre-COVID-19 times) for using public transport services if operators implemented sanitising measures.

As Table 3 shows, the main measures demanded among public transport users to use this mode are increasing supply to avoid crowding (70.6%) and increasing cleanliness and sanitising (52.1%). These findings may indicate that these measures seem to be enough to keep pre-COVID-19 levels in public transport demand and that citizens reasonably trust sanitising processes conducted under public transport authorities. It is worth noticing that 52.9% and 40.0% of these individuals would pay more, respectively, if operators implemented additional supply and sanitising actions. These results may indicate that individuals would not perceive such a dangerous option in public transport (in terms of sanitary conditions), or that they are captive of this transport mode and would use it in any case.

The willingness to use bike-sharing or kick scooter-sharing is reasonably high, around 67.7% among the sample (Table 2), which seems somewhat surprising given that demand for these transport alternatives was marginal in Spain in pre-COVID-19 times. This result may be explained by the fact that individuals find more comfortable or safer transport options that provide an open environment. Nevertheless, the willingness to pay particular measures against COVID-19 is relatively low: only 36.4%. The measures more demanded by people (Table 3) are, by far, the provision of covers for handlebars and steering wheels (51.3%), and the provision of masks, gloves, and sanitiser gel (38.1%). However, only 36.4% and 27.5% potential user would pay more.

The willingness to use taxi/ride-hailing services is in the same order of magnitude (66.4% of total respondents) than bike-sharing or kick scooter-sharing. We should note that 76.3% of people would use these services would demand increasing cleanliness and sanitisation, and 48.9% of them would pay more for it. Lower but noticeable percentages are observed for additional measures such as providing masks, gloves, and sanitiser gel before each use.

Similarly to taxi/ride-hailing, data for car-sharing reinforces the importance given by individuals to the increase of cleanliness and sanitising and the provision of masks, gloves, and sanitiser gel. These measures seem essential for transport options involving closed spaces and operated by private companies.

Tables 2 and 3 also include the distribution of survey responses concerning other transport modes addressed in the questionnaire, such as moped scooter-sharing. Comparatively lower positive responses (albeit noticeable) regarding this transport can be interpreted by the fact that it was a marginal transport option in pre-COVID-19 times in Spain. Moped scooter-sharing follow the same trend observed for bike-sharing/kick scooter-sharing, regarding the importance of providing covers for handlebars and steering wheels, masks, gloves sanitiser gel. Additionally, it is also relevant for this transport option to provide helmets with no contact with mouth, nose, and eyes (38.5% of potential users would demand this measure).

These trends should be observed in parallel with the potential influence that socio-demographic and mobility attributes may have on individuals’ responses (Table 2). Due to length limitations, only the most noticeable trends are commented. For instance, a noticeable higher proportion of females (75.4%) were willing to use taxi/ride-hailing services in post-COVID-19 times, compared to males (66.4%), which would reflect females’ preference towards ‘private’ modes. Additionally, as seems reasonable willingness to use bike-sharing, kick scooter-sharing, moped scooter-sharing, and car-sharing decreases with age, which is consistent with previous literature on shared mobility (see, e.g. [3]).

Similarly, the effect of age seems to be behind the fact that students present a higher willingness to use ride-hailing, car-sharing, and moped scooter-sharing options compared to other occupations. Finally, individuals who lost their job with COVID-19 lockdown reported lower willingness to pay for using specific transport modes, such as public transport or car-sharing, compared to the general sample. For instance, 40.0% of these individuals declared that they would not be willing to pay more for using public transport if additional sanitising actions would be implemented, compared to the percentage observed for the global sample (25.4%). This result is presumably explained by the loss of purchasing power generated by the pandemic for this segment of individuals.

It is also remarkable that demanding operators policies to only accept recently COVID-19-negative tested users while using their services are the least demanded option for every transport mode. However, potential users of car-based transport modes are more demanding: their willingness to use is 26.3% for car-sharing and 19.9% for taxi/ride-hailing, but only 17.6% and 13.6% would pay more for these kinds of measures.

**Fig. 4:**
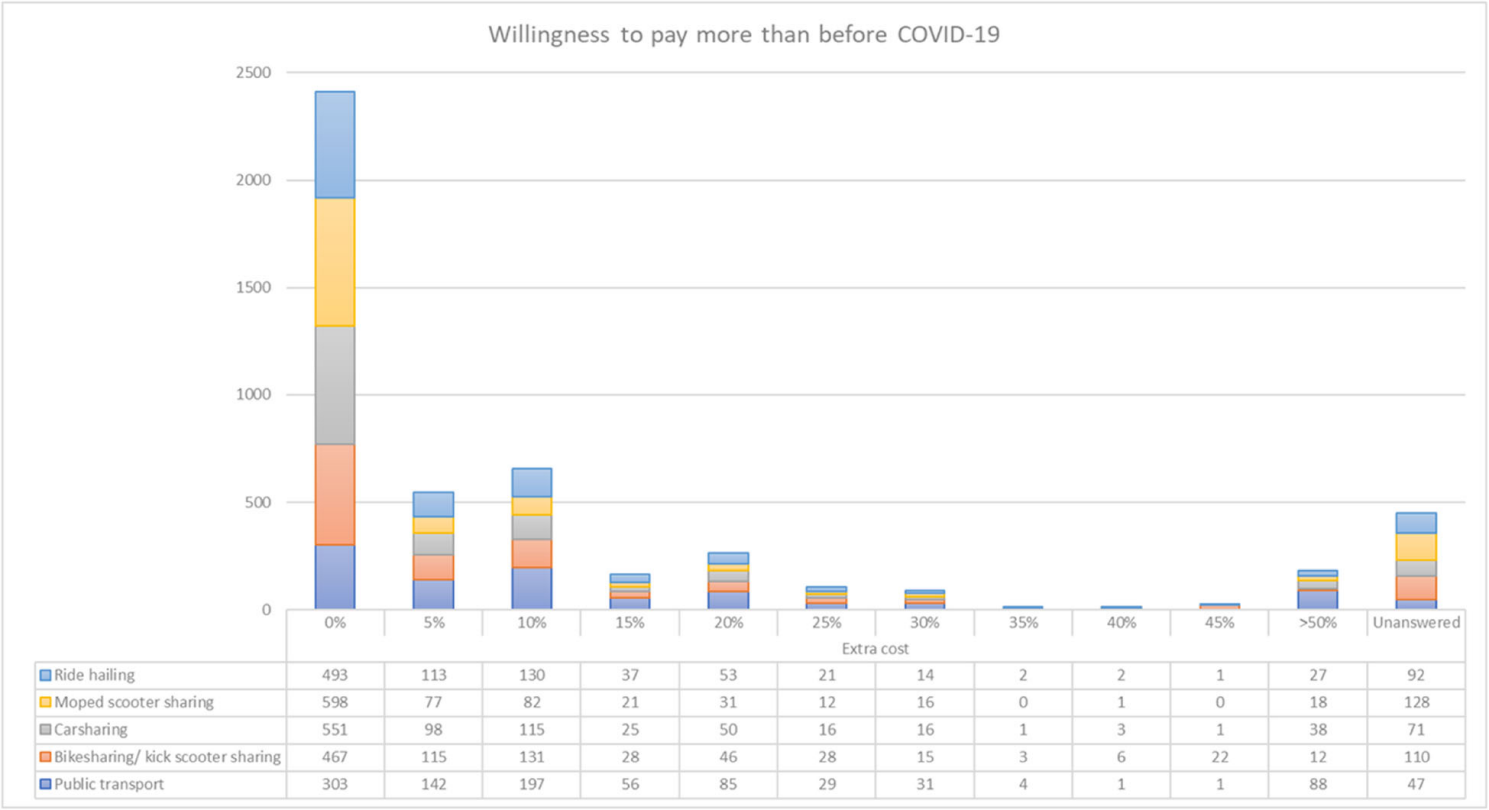
Willingness to pay more for special sanitising measures, compared to pre-COVID-19

Finally, there is a general reluctance to pay more, i.e., the low willingness to pay, for all the means of transport when respondents were also asked how much more they would pay at the service’s current price because of implementing those measures (Fig. 4). Interestingly, the most significant willingness to pay is perceived in public transport, even to a large extent accepting surcharges above 50%. This result is striking since it is the mode that would expect a higher decrease in the number of passengers. This high WTP occurs mostly among those who would be willing to use public transport if its frequency were increased and those who would do so if vehicles were cleaned and disinfected daily. This result shows that those who will continue to use it know this type of measure’s high economic cost.

### Selection equation: willingness to use a specific transport mode in post-COVID-19 times

A Heckman choice framework has been adopted to analyse individuals’ responses more rigorously. Explanatory variables used in the models were mostly categorical, so a base reference has been chosen in each case as referred to in Tables 4 and 5 when necessary. Multiple tests conducted for checking the presence of a strong correlation among the explanatory variables showed no multicollinearity problems in our data.

**Table 4:** Modelling results, Heckman model: willingness to pay (eq. 2: outcome) to use a specific transport mode (ordered probit equation)

EQUAT.	*VARIABLES (Base reference)*				PUBLIC TRANSPORT			CAR-SHARING			BIKE-SHARING / KICK SCOOTER-SHARING			TAXI / RIDE-HAILING			MOPED SCOOTER-SHARING	
					Coeff.	p-value		Coeff.	p-value		Coeff.	p-value		Coeff.	p-value		Coeff.	p-value
**WILLINGNESS TO PAY**	SOCIODEMOGRAPHICS	*Gender (male)*																
			Female															
		*Lost employment after COVID*			−0.319	0.056		−0.573	0.000		−0.163	0.033						
	MOBILITY HABITS	*Commuting (base case: intensive, public transport/sharing)*																
			Intensive, walking					0.303	0.015									
			Intensive, private bike								−0.987	0.068						
			Non-intensive, public transport		−0.508	0.036		−0.775	0.018					−0.608	0.022			
			Non-intensive, private bike								−1.260	0.008						
		*Leisure (base case: intensive, public transport/sharing)*																
			Intensive, private car/moto											0.213	0.058			
			Intensive, sharing		0.649	0.005												
			Non-intensive, private bike								0.421	0.004						
			Non-intensive, walking											0.314	0.054			
		*Shopping (base case: intensive, public transport/sharing)*																
			Intensive, public transport								−0.668	0.003						
			Intensive, private car/moto								−0.743	0.053		0.231	0.069			
			Intensive, walking		0.199	0.019					−0.206	0.048						
			Non-intensive, public transport															
			Non-intensive, private car/moto								−3.922	0.046						
			Non-intensive, walking		0.245	0.009												
	MEASURES TO PREVENT AGAINST COVID	*Increasing supply to avoid crowding*			0.192	0.018								0.247	0.027			
		*Increasing cleanliness and sanitising*									−0.580	0.060		0.321	0.001			
		*Providing masks, gloves and sanitiser gel*			0.125	0.099					0.202	0.035						
		*Certifying that only those non infected by COVID can use the service*												0.373	0.000		0.269	0.110
		*Certifying that no user within the same day was infected by COVID*									0.251	0.047						
		*Providing helmets with no contact with mouth, nose and eyes*									0.191	0.076						
		*Increasing bike-related infrastructure*									0.495	0.096						
		*Providing covers for handlebars and steering wheels*									0.341	0.000						
		Constant			2.394	0.000		0.242	0.043		0.922	0.000		0.598	0.000		−0.109	0.925
No. Obs							833			851			840			833		767
Selected observation							749			461			568			581		286
Log-likelihhod							− 1685.6			− 1409.9			− 1517.6			− 1485.7		− 974.8
Wald Chi2							65.90			25.72			58.61			47.76		14.83

**Table 5:** Modelling results, Heckman model: willingness to use (eq. 1: selection) a specific transport mode

EQUAT.	*VARIABLES (Base reference)*			PUBLIC TRANSPORT		CAR-SHARING		BIKE-SHARING / KICK SCOOTER-SHARING		TAXI / RIDE-HAILING		MOPED SCOOTER-SHARING	
				Coeff.	p-value	Coeff.	p-value	Coeff.	p-value	Coeff.	p-value	Coeff.	p-value
**WILLINGNESS TO USE**	SOCIODEMOGRAPHICS	*Monthly Income (below 500 Euro)*											
			500 to 1000 Euro										
			1000 to 1500 Euro										
			1500 to 2000 Euro							0.253	0.010		
			2000 to 3000 Euro							0.253	0.010		
			Above 3000 Euro	−0.496	0.004			−0.255	0.093				
		*Gender (male)*											
			Female	0.221	0.098					0.231	0.019		
		*Age (under 30)*											
			30 to 49			−0.381	0.000	−0.388	0.003	−0.246	0.033	−0.360	0.005
			50 to 64			−0.280	0.018	−0.613	0.000	−0.341	0.014	−0.535	0.000
			Above 64			−0.280	0.018	−1.337	0.000			−0.535	0.000
		*Occupation (employed, no teleworking)*											
			Student							0.329	0.032	0.368	0.014
	MOBILITY HABITS	*Commuting (base case: intensive, public transport)*											
			Intensive, private car/moto	−0.878	0.000								
			Intensive, bike	−1.020	0.000	−0.233	0.071			−0.243	0.094		
			Intensive, walking	−0.759	0.001	−0.211	0.095						
			Intensive, other	−1.380	0.000								
			Non-intensive, public transport										
			Non-intensive, private car/moto									−1.243	0.008
		*Leisure (base case: intensive, public transport)*											
			Intensive, private car/moto	−0.326	0.096								
			Intensive, bike	−0.587	0.015								
			Intensive, walking	−0.482	0.015								
			Non-intensive, public transport			−0.249	0.042	−0.325	0.035	− 0.256	0.078		
			Non-intensive, private car/moto	−0.431	0.058					−0.241	0.079		
			Non-intensive, walking	−0.592	0.021								
			Non-intensive, other					0.850	0.051			0.760	0.058
		*Shopping (base case: intensive, public transport)*								−0.800	0.028		
			Intensive, private car/moto							0.288	0.050		
			Non-intensive, private car/moto	−0.441	0.004								
		*Valuing comfort*				0.068	0.013			0.088	0.048		

Table 4 shows the results for the ordered probit equation (willingness to pay, eq. 2: outcome), while results for individuals’ willingness to use a specific transport mode in post-COVID-19 times (eq. 1: selection) are included in Table 5 and commented below. Most of the explanatory variables that resulted in non-statistically significant were finally removed from the last version of the model with no impact in the overall fitting, as confirmed by multiple likelihood-ratio (LR) tests conducted.

The results for the selection equation confirm what was observed in the preliminary findings. Regarding socio-demographic attributes, we can observe that as age increases, individuals show a statistically significantly lower willingness to use car-sharing, bike-sharing/kick scooter-sharing, taxi/ride-hailing and moped scooter-sharing. Since these modes typically require the use of a smartphone, this result seems reasonable given the lower tech-savviness among older segments of the population (see, e.g., [24]. Additionally, modes such as shared bikes or mopeds require being in good physical condition (see, e.g., [2]). The positive and statistically significant results obtained for students regarding taxi/ride-hailing and moped scooter sharing can be interpreted in the same line.

Regarding gender, the higher likelihoods observed for females to use public transport and taxi/ride-hailing options have been widely referred to in the literature. Additionally, according to the modelling results, individuals with higher income levels typically present a statistically significant higher willingness to use taxi/ride-hailing services and a lower willingness to use public transport, bike-sharing, or kick-scooter sharing. This result can be explained by the higher car prone attitude of wealthy individuals and their tendency towards separating or differentiating from others as a signal of exclusivity as noted by Chevalier & Gutsatz [11].

Regarding mobility habits, the most noticeable results concern the lower likelihood to use public transport services in post-COVID-19 times among individuals who commuted intensively by using any other modes: private vehicle, personal bike, walking, shared modes, among others. Nevertheless, we should remind that overall willingness to use public transport services in post-COVID-19 times was high in the sample, as noted above. Detailed results concerning the frequency of use regarding any trip purpose and transport mode can be observed in Table 5.

### Ordered probit equation: willingness to pay more to use a specific transport mode in post-COVID-19 times

Table 4 includes the modelling results regarding the Heckman regression equation (willingness to pay, eq. 2: outcome). As can be observed, socio-demographic variables play a minor role in explaining individuals’ willingness to pay. Only statistically significant results were found for individuals losing their job after the beginning of the COVID-19 lockdown regarding car-sharing (*p*-value = 0.000), scooter-sharing (p-value = 0.033), and public transport (p-value = 0.058), which is reasonable given the loss in the purchasing power of this segment of the population. Other socioeconomic variables such as gender or age did not result statistically significant for transport services analysed.

Regarding explanatory variables controlling for mobility habits, many coefficients appeared as statistically significant. Mainly, individuals who commute (intensively or not) by private bike present a lower willingness to pay for using bike-sharing systems, which seems evident given that using their own bike is cheaper and safer against contagion. Nevertheless, the opposite effect is observed for those who use their private bike non intensively for leisure trips (*p*-value = 0.008). Additionally, occasional users of public transport for commuting purposes showed a statistically significant lower willingness to pay for additional costs for sanitary measures on public transport (*p*-value = 0.036), car-sharing (*p*-value = 0.018) and hailing (p-value = 0.022) services, in case an additional cost was imposed to the user for implementing sanitation measures. Detailed results concerning the influence of mobility habits on individuals’ willingness-to-pay can be observed in Table 4.

More interestingly, Table 4 includes modelling results controlling for willingness to pay in specific modes of transport and potential measures to be implemented during post-COVID-19 periods. Reasonably, increasing service supply to avoid crowding (p-value = 0.018) and, to a lower extent, providing masks, gloves, and sanitiser gel (*p*-value = 0.099) would increase individuals’ willingness to pay for using public transport. Together with the preliminary findings above, these results are significant given the additional costs these measures imply to operators and current financing problems on public transport services. By contrast, no measures are statistically significant for car-sharing options, although one may expect that, e.g., improving cleanliness and sanitising or providing covers for handlebars and steering wheels, would increase willingness-to-pay for these services. Thus we can conclude that car-sharing users do not value sanitising measures in such a way to lead to a higher willingness to pay.

Regarding hailing services (taxi/ride-hailing), typically with higher unitary costs per km, we can observe that willingness to pay would be higher in a statistically significant way among users demanding higher supply (*p*-value = 0.027), increase of cleanliness and sanitising (p-value = 0.001), and a certification that only those non infected by COVID can use the service (p-value = 0.000). These results can be explained in the light of the central aspect: individuals with a higher purchasing power typically use ride-hailing services, therefore more open to paying more for certain services.

Similar to the findings on public transport, for bike-sharing/kick scooter-sharing we can observe (Table 4) that several measures could lead to a higher willingness-to-pay, namely: providing masks, gloves, and sanitiser gel (p-value = 0.035); certifying that no user within the same day was infected by COVID (p-value = 0.047); providing helmets with no contact with mouth, nose, and eyes (p-value = 0.076) or providing covers for handlebars and steering wheels (p-value = 0.000). Surprisingly, cleanliness and sanitising actions negatively influences the willingness to pay for these services. One may conclude that this type of measure is viewed by individuals as prerequisites for using a transport services in post-COVID-19 times, but not necessarily an element to lead to a higher willingness to pay, despite the greater costs it imposes on operators. Additionally, it seems that bike-sharing users demanding the improvement of bike infrastructure (e.g., extending current bike lanes) would be willing to pay more in a statistically significant way since this result is close to being statistically significant (p-value = 0.096).

Finally, modelling results for moped scooter-sharing present many similarities with car-sharing since almost no measure increases willingness-to-pay for these services. The only exception would be certifying that only individuals non infected by COVID-19 could use this service, but this result is not statistically significant (p-value = 0.110). Thus we can again conclude that, despite the higher costs that sanitising measures impose on operators, they are not valued by users of moped scooter-sharing in such a way to result in a higher willingness to pay. On the contrary, are seen as prerequisites for using the service.

## Conclusions

Because of the COVID-19 pandemic, governments had to deploy severe measures to contain the virus spread. In the case of Spain, the almost three-month lockdown reduced most of the economic activities to a halt. After this situation, with the new normality, economic activities have not recovered the performance of pre-COVID-19 situation: some people have lost their jobs, others are teleworking, studying from home, among others. In this way, people’s mobility has changed, both in the frequency of travel and trip purposes.

With the hypothesis that the fear of contagion could also have been a reason to change travel behaviour, given that people would opt more for individual modes of transport, a survey has been carried out to find out which measures can help public transport and shared mobility services to be used. Similarly, this work is intended to provide an overview of users’ willingness to pay if the incorporation of these measures would entail an extra cost for them. The results are particularly relevant given that profit margins in the transport sector are very low in Spain or, in many cases, public services have to be subsidised because they are loss-making.

According to the survey findings, the general willingness to use different modes of transport in the post-COVID-19 period varies greatly. The set of measures that would help respondents accept or not use each mode of transport after the lockdown period is detailed in Table 6.

**Table 6:** Classification of measures according to their high/low effect on WTU

MEAN OF TRANSPORT	HIGH EFFECT ON WTU	LOW EFFECT ON WTU
PUBLIC TRANSPORT	Increasing the frequency of the service to avoid crowding	Free masks, gloves, or hydroalcoholic gels provided with each use
	Intensive vehicle cleaning	If only those not contracting/positive for COVID-19 are certified to use the service
CAR-SHARING	Intensive sanitising of the vehicles	Increasing supply to avoid crowding
	Steering wheel covers	Free masks, gloves, or hydroalcoholic gels provided with each use
		If only those not contracting/positive for COVID-19 are certified to use the service
BIKE-SHARING / KICK SCOOTER-SHARING	Free masks, gloves, or hydroalcoholic gels provided with each use	
	Handlebar covers	If only those not contracting/positive for COVID-19 are certified to use the service
	Free masks, gloves, or hydroalcoholic gels provided with each use	
TAXI / RIDE-HAILING	Intensive sanitising of the vehicles	Increasing supply to avoid crowding
		Free masks, gloves, or hydroalcoholic gels provided with each use
		If only those not contracting/positive for COVID-19 are certified to use the service
MOPED SCOOTER-SHARING	Free masks, gloves, hydroalcoholic gels, and surgical scrub caps to avoid direct contact with the helmets^a^ provided with each use	If only those not contracting/positive for COVID-19 are certified to use the service
	Handlebar covers	

^a^Should be positive to promote the visibility of this kind of special service already offered before COVID-19

Public transport is, according to respondents, the option with the highest willingness to use. 89.7% of individuals reported that they would use these services in the post-lockdown period, a figure that seems high given that the survey was carried out during the critical period of the lockdown when the demand of public transport dropped up to 40–70% of the same period of 2019 in some of the biggest Spanish cities.

The willingness to use bike-sharing or kick scooter-sharing is also relatively high (67.7%), a striking result given that the demand for these modes of transport was marginal in Spain in the pre-COVID-19 period. This result is also the case, to a lower extent, for other shared modes and taxi/ride-hailing. We recommend taking these results with caution since the willingness to use is on the side of casual trips, rather than for the most frequent ones.

Based on the results, it appears that the WTP extra costs are, however, moderate. In other words, transport is a well-established activity in Spain, and its adaptation to the new needs imposed by the pandemic seems to be taken for granted by the users, who do not consider that the value of the service adaptations requires a higher payment than in the pre-COVID-19 period. There is only one exception: taxi/ride-hailing services do seem to be understood as luxury services. This result may be due to the fact that most trips in Spain are made by public transport, by private car and by walking [2]. Taxi/ride-hailing services are rarely used and for concrete reasons where the WTP more is reasonable, such as business trips paid by companies or night-time trips made when there is less public transport available and paying for the taxi/ride-hailing service means a significant saving of time to return home after leisure or work.

For public transport, the measures most widely accepted are those related to increasing the frequency of the service to avoid crowding and intensive vehicle cleaning. In other words, those that are the most expensive ones. Given the additional costs that these measures imply for operators and the current problems of financing transport services, this resistance to payment can be problematic for the finance structure of the transport system.

For sharing services, the intensive sanitising of the vehicles should be continuously advertised by operators, as it is a crucial measure for the users among all the shared modes. Scooter-sharing services already offered special gear such as surgical scrub caps to avoid direct contact with the helmets before the pandemic. It is recommended to promote the visibility of these measures, also, to offer extra protection such as disposable gloves, steering wheel/handlebar covers and hydroalcoholic gel. The same as with public transport, the implementation of the measures are taken for granted. Users do not seem willing to have the cost of these measures passed on to them, which is a problem for operators because operating costs must be increased without affecting tariffs.

This result is an exciting approach for researchers interested in more in-depth analysis. Nevertheless, it also allows to highlight the importance of increasing public funding of the public transport system or establishing new indicators of charging operators since some public transport companies in Spain charge per passenger. Thus, the mechanisms for charging per kilometre or per passenger-kilometre make it possible to support the system better. Given that Spain lacks a law on the financing of public transport, the current situation might be a catalyst to undertake this project in brief to avoid the arising of financial problems for transport operators. It is recommended that this funding law should prioritise investment in public transport and the improvement of soft modes rather than sharing and taxi/ride-hailing services. In case of the latter are to be financed, their services will have to be regulated in a way that subsidies ensure that services can be used on equal terms by all users. This regulation should guarantee that public resources are equitability invested.

Finally, it is worth mentioning that this research has some limitations due to the extraordinary situation when it was conducted, which may open up future research questions. Firstly, the answers obtained in such an exceptional period may be highly conditioned by traumatic experiences during the lockdown and the high degree of socioeconomic uncertainty about the evolution of the pandemic and its consequences when it ceases. It was difficult for the respondents to predict the magnitude of the pandemic effects on day-to-day life, then the obtained responses were partially fulfilled.

It is necessary to take the results of this work with the proper caution. In this sense, it is necessary to clarify that the interaction through a survey would be completed with other sources of mobility data and future similar surveys. It is difficult to discern with complete certainty whether the results towards the different modes reflect a previous idea that is crystallising or whether, by contrast, it is the shock produced by the pandemic that produces this change in preferences.

Also, due to the short period for sending papers for this particular issue, we must say that for this paper we have focused only on the case of Spain, but the survey was carried out at an international level, obtaining responses from several Latin American countries. Therefore, in future analyses, we hope to study the differences between regions and countries. Other important outcomes could be obtained due to the different mobility alternatives and socioeconomic characteristics of the different regions.

## Data Availability

All data and materials used for the writing of this paper are the property of the researchers. The survey database is the property of the researchers. It is not yet publicly available but it is available from the corresponding author on reasonable request, and in compliance with Spanish data protection law.
